# Hand assessment in older adults with musculoskeletal hand problems: a reliability study

**DOI:** 10.1186/1471-2474-12-3

**Published:** 2011-01-07

**Authors:** Helen L Myers, Elaine Thomas, Elaine M Hay, Krysia S Dziedzic

**Affiliations:** 1 Arthritis Research UK Primary Care Centre, Keele University, Keele, Staffordshire, UK; 2 Staffordshire Rheumatology Centre, The Haywood, Burslem, Stoke-on-Trent, UK

## Abstract

**Background:**

Musculoskeletal hand pain is common in the general population. This study aims to investigate the inter- and intra-observer reliability of two trained observers conducting a simple clinical interview and physical examination for hand problems in older adults. The reliability of applying the American College of Rheumatology (ACR) criteria for hand osteoarthritis to community-dwelling older adults will also be investigated.

**Methods:**

Fifty-five participants aged 50 years and over with a current self-reported hand problem and registered with one general practice were recruited from a previous health questionnaire study. Participants underwent a standardised, structured clinical interview and physical examination by two independent trained observers and again by one of these observers a month later. Agreement beyond chance was summarised using Kappa statistics and intra-class correlation coefficients.

**Results:**

Median values for inter- and intra-observer reliability for clinical interview questions were found to be "substantial" and "moderate" respectively [median agreement beyond chance (Kappa) was 0.75 (range: -0.03, 0.93) for inter-observer ratings and 0.57 (range: -0.02, 1.00) for intra-observer ratings]. Inter- and intra-observer reliability for physical examination items was variable, with good reliability observed for some items, such as grip and pinch strength, and poor reliability observed for others, notably assessment of altered sensation, pain on resisted movement and judgements based on observation and palpation of individual features at single joints, such as bony enlargement, nodes and swelling. Moderate agreement was observed both between and within observers when applying the ACR criteria for hand osteoarthritis.

**Conclusion:**

Standardised, structured clinical interview is reliable for taking a history in community-dwelling older adults with self reported hand problems. Agreement between and within observers for physical examination items is variable. Low Kappa values may have resulted, in part, from a low prevalence of clinical signs and symptoms in the study participants. The decision to use clinical interview and hand assessment variables in clinical practice or further research in primary care should include consideration of clinical applicability and training alongside reliability. Further investigation is required to determine the relationship between these clinical questions and assessments and the clinical course of hand pain and hand problems in community-dwelling older adults.

## Background

Musculoskeletal hand pain and hand problems are common in the general population [1-4], with the hand being one of the most common sites of pain and osteoarthritis (OA) in older people [5,6]. Despite clinical history taking and physical examination being key to clinical decision-making [7-10], few studies have considered the reliability of these methods of gathering information in those with undifferentiated hand pain presenting in primary care.

A Delphi study with 26 UK Health Care Practitioners [11] identified a range of simple questions and physical examinations for use in primary care with older adults with self reported hand pain and problems. In this paper we describe the results of a reliability study in which we investigated the extent of inter-and intra-observer reliability for these, and some additional physical examination items, in a primary care population. Additionally, the reliability of applying the American College of Rheumatology (ACR) criteria for symptomatic hand OA [12] is reported.

## Methods

Ethical approval for the study was obtained from the North Staffordshire Local Research Ethics Committee (REC reference number: 02/54).

### Observers

Observers were an Occupational Therapist and a Physiotherapist with 12 and 22 years post-qualification experience respectively. A manual of detailed protocols was developed and used to train and standardise the observers prior to the study, and for reference during the study. Briefly, these protocols outlined the objective, methods, recording instructions and special notes for each question and assessment. In addition, skip patterns for questions were described, and a detailed description was provided for each assessment, supplemented by photographs to aid standardisation. Prior to the study, both observers had undergone training in research interview procedures and physical examination techniques as part of another study [13].

### Participants

The sampling framework consisted of 201 people aged 50 years and over registered with one general practice who had previously completed a postal questionnaire as part of a study of hand pain and problems in the population [14] and who fulfilled the following criteria: experienced hand pain or problems within the last 12 months (consultation was not required); completed questions on the presence of nodes and functional limitation; and consented to further contact. Exclusion criteria were accident, injury or surgery to the hands in the past month. A purposive sampling strategy based on presence of nodes and functional limitation was used to ensure that a spectrum of severity of hand problems and hand functional limitation would be represented in the study.

### Procedure

Potential participants were sent a letter of invitation and an information sheet explaining the study and were asked to telephone the research centre if they were interested in participating. Those who did were screened for eligibility and were offered an appointment at a research clinic held at their general practice.

Consenting participants were asked to attend for two appointments, one month apart. At the first appointment, both observers independently assessed each participant. Allocation of participants to observers and the order of assessment were not randomised. However, by inviting participants to attend in pairs, so that each observer saw the same number of participants first and second, the potential for order effects was reduced. Observers were blind to the results of each other's assessment and to existing data relating to participants' hand problems. Participants were asked to complete a brief self-administered questionnaire.

At the second appointment, participants were assessed by one observer and repeated the brief self-administered questionnaire. To identify self-reported changes in overall hand problems between the first and second appointment, participants were asked to report whether their hand problem was "better," "worse," or "about the same." To minimise missing data, a research nurse checked all assessment forms and questionnaires at both appointments.

### Data collection

Clinical interview questions covered aspects of hand problems such as location (one or both hands, worst hand), handedness, history and duration, specific symptoms (pain, tenderness, aching or discomfort, stiffness, locking or triggering, altered sensation), functional limitation, impact of and adaptation to hand problems, self management, and causal and diagnostic attributions.

The physical examination included a screen of upper limb movement (adapted from [15] to include radio-ulna supination and pronation, finger flexion and extension, wrist flexion and extension, and shoulder external rotation), observation of muscle wasting, observation and palpation of bony enlargement, deformity, swelling, and Dupuytren's contracture, and palpation of joint pain and tenderness. Wrist and thumb range of movement and pain on resisted movement were also measured. Specific tests were carried out: Phalen's [16,17], Grind [18,19], and Finklestein's [18,20]. Sensation was evaluated using Semmes-weinstein™ monofilaments. Grip and pinch strength were measured using a Jamar dynamometer and a B&L pinch gauge respectively [21], and hand function was assessed using the Grip Ability Test [22].

In the self-administered questionnaire, participants completed the AUStralian CANadian Osteoarthritis Hand Index (AUSCAN) [23] and answered questions about pain, stiffness and swelling in the hands and fingers, perceived hand strength, severity of hand pain (numerical rating scale), severity of hand problems and bothersome-ness of hand problems.

### Statistical analysis

To detect a Kappa of ≥ 0.5 (two-tailed α = 0.05, power = 0.95) a minimum of 52 participants were required [24]. To allow for potential drop out we aimed to recruit 60 participants.

For categorical and numerical data, two analyses were carried out: inter-observer and intra-observer (test-retest) reliability. For categorical data, inter-and intra-observer reliability was summarised using percentage prevalence (based on the average of findings from both observers), the number of cases agreed, percentage observed agreement, percentage expected agreement and Cohen's Kappa (dichotomous data) or quadratic weighted Kappa (ordinal data), with 95% confidence intervals. Where observed agreement was 100% in one direction, Kappa was not calculated. Where physical examination was carried out at multiple sites, (e.g. 19 areas per hand were palpated for pain and tenderness), data were summarised using median and range (minimum to maximum) for percentage agreement and Kappa. Analysis of dichotomous data was performed using Programs for EPIdemiologists (PEPI) version 1.15 [25], and analysis of ordinal data was carried out using Vassarstats [26]. For examinations where there was poor agreement, a comparison of the number of positives identified by the observers was carried out to explore whether any differences were due to chance or to systematic over-reporting or under-reporting. Due to the influence of high or low prevalence on Kappa, in instances where prevalence was either very high or very low, the interpretation of Kappa values was considered together with the levels of agreement.

Kappa values were categorised as "almost perfect" (Kappa > 0.80), "substantial" (0.61-0.80), "moderate" (0.41-0.60), "fair" (0.21-0.40), or "slight/poor" (≤ 0.20) [27]. For numerical data, intra-class correlation coefficients (ICC_2,1_) were calculated (two way random, absolute agreement) and were categorised as "adequate" (ICC > 0.9), "good" (0.75-0.90) or "moderate to poor" (< 0.75) [28].

In items with high observer variability (Kappa ≤ 0.61 and observer agreement below 80%) we explored two possible sources of disagreement: order effects as a source of inter-observer variability, and self-reported change in overall hand problems as a source of intra- observer variability. Order effects were investigated for each relevant item by comparing the proportion of positive findings from the first and second observer in all cases. The effect of self-reported overall change in hand problem status was explored through examination of the single transition question on the self-administered questionnaire.

## Results

Of the 56 people who met the eligibility criteria and were invited to attend, 55 (22 male: 33 female) attended the first clinical assessment. The mean (standard deviation) age was 66 (8) years. Their median (observed minimum to maximum range) AUSCAN scores for pain, stiffness and function were 8 (0-20), 1 (0-4), and 10 (0-36) respectively, suggesting moderate restriction of hand function. One participant did not attend their second appointment, leaving 54 for the analysis of intra-observer reliability.

### Clinical interview

#### Inter-observer reliability

Using the previously defined cut-offs for Kappa, agreement beyond chance for inter-observer ratings can be considered to be "almost perfect" for seven of the questions, "substantial" for ten, "moderate" for seven, "fair" for one and "slight/poor" for one (Table 1).

**Table 1:** Reliability of questions asked during clinical interview and self-complete questionnaire (in order of agreement beyond chance for inter-observer comparisons)

	Inter-observer reliability (*n *= 55)				Intra-observer reliability (*n *= 54)		
	
	Prev %	Agr	Agr %	*K*	Agr	Agr %	*K*
Do you have problems with one or both hands? *(left/right/both*^+^*)*	84.6^+^	5/3/46	98.2	0.93	3/3/43	90.8	0.67
Have you ever had surgery to your hands? *(yes/no)*	15.5	8/46	98.2	0.93	8/45	98.1	0.93
Have you ever had injuries to your hands? *(yes/no)*	49.1	25/26	92.7	0.85	26/26	96.3	0.93
Are you right or left handed? *(right*^+^*/left/both)*	94.5^+^	52/2/0	98.1	0.83	51/3/0	100	1.00
Does your hand pain stop you from sleeping or disturb your sleep? *(yes/no/na)*	30.9	15/29/4	92.7	0.83	12/23/5	87.0	0.68
How long does hand stiffness last on waking? *(< 30 mins^+^≥30 mins/na)*	46.4^+^	23/6/18	91.0	0.82	18/4/15	74.1	0.48
When did your hand symptoms first start? *(< 12 mnths*^+^*/1-5 yrs/5-10 yrs/> 10 yrs)*	8.2^+^	3/20/6/19	87.3	0.81	1/17/3/20	76.0	0.63
Do you ask for help? *(yes/no)*	66.4	34/16	90.9	0.80	37/13	92.6	0.82
Do your fingers ever lock, trigger or catch? *(yes/no)*	23.7	11/40	92.7	0.80	11/39	92.6	0.80
Which hand is worse? *(left/right*^+^*/no diff/na)*	48.2^+^	10/24/5/8	85.4	0.79	7/21/5/6	72.3	0.60
Do you have pins and needles in your hands? *(yes/no)*	45.5	22/27	89.1	0.78	17/24	75.9	0.51
Do you have hand stiffness on waking? *(yes/no/na)*	61.8	31/6/8	89.1	0.77	29/2/9	81.5	0.60
Do you have numbness in your hands? *(yes/no)*	30.9	14/35	89.1	0.75	10/36	85.2	0.62
Is your hand pain there all the time? *(yes/no/na)*	30.9	14/27/4	89.1	0.75	14/17/5	75.9	0.49
Is the altered sensation worse at night? *(yes/no/na)*	22.8	9/17/20	87.3	0.64	6/10/19	75.9	0.33
Have you had to stop work, chores or hobbies because of your hand symptoms? *(yes/no)*	30.2	12/34	83.6	0.61	11/32	79.6	0.52
Do you experience stiffness in your hands? *(yes/no)*	78.2	39/8	85.4	0.57	36/9	83.4	0.57
Do you use gadgets or aids? *(yes/no)*	50.9	22/21	78.2	0.57	21/21	77.8	0.56
In the last month have you had any pain, tenderness, aching or discomfort in your hands? *(yes/no)*	88.2	46/4	90.9	0.57	41/5	85.2	0.47
Have you changed the way you do things? *(yes/no)*	74.6	36/9	81.9	0.53	32/4	66.7	0.09
Does hand pain limit your activities? (*yes/no/na)*	39.1	15/21/4	76.4	0.52	14/21/5	83.3	0.63
Have you had to take time off work because of your	8.2	2/6/35	78.2	0.51	1/4/41	85.2	0.57
hand problem? *(yes/no/retired)*							
Do you have tingling in your hands? *(yes/no)*	39.1	13/29	69.1	0.35	9/27	66.7	0.26
Do you have any other sensation in your hands?	6.4	0/48	87.3	-0.03	0/52	96.3	-0.02
*(yes/no)*							
Do you experience any thumb pain during activity? *(yes/no)*^§^	-	-	-	-	45 (30/15)	83.3	0.64
Severity of hand problems *(none/very mild/mild/moderate/severe)*^§^	-	-	-	-	33 (1/6/10/13/3)	61.2	0.57^
Days of pain, aching, stiffness in the last month *(no/few/some/most/all)*^§^	-	-	-	-	29 (2/5/8/7/7)	53.8	0.56^
Bothersomeness *(not at all/slightly/moderately/very much/extremely)*^§^	-	-	-	-	31 (3/10/14/3/1)	57.5	0.53^
Symptom progression *(worse/better/same)*^§^	-	-	-	-	34 (5/0/29)	63.0	0.19^
		**Agr % Median (range)**	***K *Median (range)**		**Agr % Median (range)**	***K *Median(range)**	
Can you show me where you have altered sensation? (Hand manikin - 14 areas per hand)	-	90.0 (83.6, 100.0)	0.66 (0.20, 1.00)		83.3 (74.1, 90.7)	0.47 (-0.09, 0.65)	
Can you show me where your hands hurt? (Hand manikin - 14 areas per hand)	-	85.5 (76.4, 94.5)	0.58 (0.27, 0.75)		84.3 (75.9, 90.7)	0.43 (0.05, 0.73)	

Prevalence % is based on the average of findings from both assessors. Percentages are based on positive findings unless otherwise indicated^+^. Abbreviations: Prev % = prevalence of positive findings based on first assessor; Agr = Number of cases agreed; Agr % = observed percentage agreement; k = kappa; ^quadratic weighted kappa; ^§^Questions from self-complete questionnaire; *Median (range) for observed percentage agreement and kappa.

#### Intra-observer reliability

Agreement beyond chance was lower for intra-observer than for inter-observer ratings: Kappa values can be considered to be "almost perfect" for four questions, "substantial" for six, "moderate" for 12, "fair" for two and "slight/poor" for two.

#### Self-administered questionnaire

Test-retest reliability for self-administered questions ranged from "slight/poor" (K = 0.19) to "substantial" (K = 0.64) (Table 1), with questions relating to swelling and thumb pain having the highest Kappa values. The reliability of the pain numerical rating scale was "moderate to poor" (K = 0.59).

### Clinical assessment

#### Agreement for individual hand assessment variables

For inter-observer ratings, Kappa values were "almost perfect" for one item, "substantial" for one, "moderate" for five, "fair" for three, and "poor" for one (Table 2). For intra-observer ratings, Kappa values were "almost perfect" for two of the assessments, "substantial" for one, "moderate" for six, "fair" for one, and "poor" for one.

**Table 2:** Reliability of hand assessment variables

	Inter-observer reliability (*n *= 55)				Intra-observer reliability (*n *= 54)		
	
	Prev %	Agr	Agr %	*K*	Agr	Agr %	*K*
Dupuytren's - L *(present/absent)*	17.3	8/44	94.5	0.81	6/45	94.4	0.77
Hand function: Grip Ability Test (GAT) *(score grouped into quintiles*^‡^*)*	-	(7/4/6/5/10)	58.2	0.62	(6/4/3/4/9)	48.1	0.54
Phalen's test - R *(pos/neg/uta)*	29.1	11/33/1	81.8	0.58	12/30/1	79.6	0.56
Finklestein's test - L *(pos/neg/uta)*	9.1	3/48/0	92.7	0.56	3/45/0	88.9	0.45
Grind test - R *(pos/neg/uta)*	18.2	6/41/0	85.5	0.51	5/42/0	87.0	0.54
Phalen's test - L (pos/neg/uta)*	18.2	5/39/1	81.8	0.49	6/34/1	75.9	0.41
Dupuytren's - R (present/absent)*	20.0	6/38	81.5	0.46	6/44	92.6	0.91
Finklestein's test - R *(pos/neg/uta)*	10.9	3/45/0	87.3	0.40	1/46/0	87.0	0.25
Grind test - L *(pos/neg/uta)*	16.4	4/41/0	81.8	0.34	8/38/0	85.2	0.57
Skin condition *(normal/discolouration)*	79.1	37/5	76.4	0.31	46/1	87.0	0.17
Global impression of upper limb *(normal/abnormal)*	81.9	37/2	71.0	0.14	52/2	100.0	1.00

Abbreviations: pos = positive test; neg = negative test; uta = unable to assess; R = right; L = left; Prev % = prevalence percentage; Agr = number of cases agreed; Agr % = observed percentage agreement; *missing data for inter-observer reliability; ^‡^the distribution of the GAT scores was highly skewed and the scores were therefore converted into quintiles and analysed using quadratic weighted Kappa. Prevalence % based on mean positive findings of both observers at 1^st ^appointment.

Preliminary analysis showed the distribution of GAT scores to be highly skewed towards lower values. As this skewed distribution remained after transformation, the data were converted into quintiles and analysed using quadratic weighted Kappa. Kappa was "substantial" (K = 0.62) for inter-observer ratings and "moderate" (K = 0.54) for intra-observer ratings (Table 2).

#### Agreement for hand assessment variables (summarised from assessments at multiple sites)

For all movements comprising the upper limb function screen, median Kappa for inter-observer ratings was "substantial" (K = 0.65) (Table 3). Of particular note was radio-ulna pronation and supination where Kappa was "fair" or "slight/poor" (data for individual movements not shown). Median intra-observer reliability of the movements comprising the upper limb function screen was similar to that seen for inter-observer (K = 0.69).

**Table 3:** Reliability for hand assessment variables (summarised from assessments at multiple sites)

	Inter-observer reliability (*n *= 55)		Intra-observer reliability (*n *= 54)	
	
	Agr % Median (range)	*Kappa *Median (range)	Agr % Median (range)	*Kappa *Median (range)
Upper limb function^1^	89.1 (80.0, 100.0)	0.65 (0.00, 0.91)	91.6 (79.6, 100)	0.69 (0.00, 0.94)
Pain/tenderness^2^	94.5 (78.2, 100.0)	0.64 (0.22, 1.00)	96.3 (85.2, 100.0)	0.69 (-0.02, 1.00)
Deformity^3^	91.8 (54.5, 100.0)	0.51 (-0.03, 1.00)	96.3 (85.2, 100.0)	0.69 (-0.02, 1.00)
Thumb opposition*^4^	98.2 (72.7, 100.0)	0.51 (0.00, 1.00)	98.1 (81.1, 100.0)	0.19 (0.00, 1.00)
Any bony change^^3^	81.8 (63.6, 98.2)	0.43 (0.00, 0.72)	83.3 (63.0, 100.0)	0.47 (0.00, 0.79)
Muscle wasting^5^	80.0 (72.7, 85.5)	0.28 (0.00, 0.38)	88.9 (81.5, 98.1)	0.29 (0.00, 0.65)
Bony enlargement^3^	81.8 (60.0, 98.2)	0.20 (-0.10, 0.68)	84.3 (61.1, 100.0)	0.30 (-0.03, 0.66)
Sensation^6^	61.8 (47.3, 70.9)	0.18 (0.04, 0.38)	76.9 (61.1, 85.2)	0.31 (0.07, 0.68)
Pain on resisted movement^7^	68.2 (61.8, 72.7)	0.16 (0.02, 0.23)	82.4 (74.5, 85.2)	0.31 (0.22, 0.50)
Nodes^3^	98.2 (65.5, 100.0)	0.00 (-0.07, 0.69)	96.3 (68.5, 100.0)	0.24 (-0.05, 1.00)
Swelling^2^	98.2 (85.5, 100.0)	0.00 (-0.03, 0.66)	99.1 (87.0, 100.0)	0.00 (-0.07, 1.00)

Abbreviations: Agr % = observed percentage agreement. *Calculated on 53 cases for intra observer reliability; ^any bony change is defined as bony enlargement or nodes. ^1^Comprising seven movements per upper limb, adapted from Doherty et al., (1992); ^2^Nineteen joints/areas per hand; ^3^Sixteen joints/areas per hand; ^4^Kapandji (1992); ^5^Four areas per hand; ^6^Semmes-Weinstein™ monofilaments used to assess median, radial and ulna nerve areas; ^7^wrist flexion/extension and thumb flexion/extension.

Median Kappa was "fair" for both inter- and intra-observer ratings of muscle wasting (K = 0.28 and 0.29 respectively), (Table 3).

Median Kappa for inter-and intra observer ratings of deformity, bony enlargement, nodes, swelling and pain/tenderness was below 0.60 for all but 3 of these items (inter- and intra-observer pain, and intra-observer deformity) (Table 3).

Median Kappa was "moderate" for inter-observer ratings of thumb opposition and "slight/poor" for intra-observer ratings. For inter- and intra-observer reliability, some ratings showed "perfect" agreement beyond chance (Table 3).

Median Kappa values for inter- and intra-observer ratings of assessment of pain on resisted movement were 0.16 ("slight/poor") and 0.31 ("fair") respectively (Table 3). A similar pattern was seen for assessment of sensation, with median inter- and intra-observer Kappa values reflecting "slight/poor" (K = 0.18) and "fair" (K = 0.31) agreement beyond chance respectively.

#### Agreement for numerical variables

Intra-class correlation co-efficients for inter- and intra-observer measurement of thumb extension, wrist extension and wrist flexion (Table 4) can be considered "moderate to poor" for seven measurements, and "good" for five measurements. The lowest ICCs (0.33 to 0.56) were obtained for measurement of thumb extension.

**Table 4:** Reliability of hand assessment variables (numerical)

	Inter-observer reliability (*n *= 55) ICC (95% CI)		Intra-observer reliability (*n *= 54) ICC (95% CI)	
	
*Variable*	Right hand	Left hand	Right hand	Left hand
Tripod pinch	0.94 (0.90, 0.97)	0.93 (0.87, 0.96)	0.93 (0.89, 0.96)	0.93 (0.89, 0.96)
Gross grip	0.91 (0.85, 0.95)	0.93 (0.85, 0.96)	0.96 (0.93, 0.98)	0.96 (0.91, 0.98)
Pulp pinch	0.89 (0.82, 0.94)	0.89 (0.81, 0.93)	0.92 (0.86, 0.95)	0.90 (0.84, 0.94)
Key pinch	0.88 (0.81, 0.93)	0.87 (0.79, 0.92)	0.88 (0.80, 0.93)	0.89 (0.81, 0.93)
Wrist flexion	0.72 (0.57, 0.83)	0.80 (0.69, 0.88)	0.75 (0.61, 0.85)	0.79 (0.67, 0.87)
Wrist extension	0.71 (0.43, 0.85)	0.70 (0.54, 0.81)	0.84 (0.73, 0.90)	0.80 (0.67, 0.88)
Thumb extension	0.46 (-0.09, 0.72)	0.56 (0.34, 0.72)	0.38 (0.14, 0.59)	0.33 (0.07, 0.55)

Abbreviations: ICC = Intraclass correlation coefficient; 95% CI = 95 percent confidence interval.

Intra-class correlation co-efficients for grip and pinch measurements ranged from 0.87 to 0.96 (Table 4). In summary, for inter-observer ratings four measurements can be considered "adequate" and four "good", and for intra-observer ratings, six of the measurements can be considered "adequate" and two "good".

#### Agreement for clinical classification

Using the ACR clinical criteria for hand OA, Kappa values reflected moderate agreement above chance for both inter- and intra-observer ratings (K = 0.43, and 0.47 respectively).

#### Sources of disagreement

No obvious systematic differences or protocol deviations emerged from post-analysis discussion to explain areas of poor inter- or intra-observer reliability. However, further analysis of disagreements between observers showed that for some assessments, namely observation of joint deformity, bony enlargement and nodes, there was a systematic difference, with one observer recording more positive findings than the other.

#### Order effects as a source of inter-observer variability

Using the previously described criteria, four clinical interview questions (do you use gadgets or aids, does hand pain limit your activity, have you had to take time off work because of your hand problem, and do you have tingling in your hands) and four clinical assessment items (assessment of skin condition, global impression of upper limb, pain on resisted movement, and assessment of sensation) showed poor inter-observer reliability (agreement < 80% and Kappa < 0.61). There were no significant differences in the proportion of positive findings between the first and second observer, suggesting that a simple order effect was not a major source of variability.

#### True change in participant status as a source of intra-observer variability

Forty-one (75.9%) of the 54 participants rated their hand problem "about the same" at the second visit when compared to their first visit a month earlier. Four (7.4%) rated their hand problems as "somewhat better" and nine (16.7%) as "somewhat worse". These numbers were too small to allow separate analysis of variability in "stable" participants but do raise the possibility of true change in participant status as a significant factor underlying intra-observer variability. For the four clinical interview questions and four clinical assessment items (previously described) showing high intra-observer variability (agreement < 80% and Kappa < 0.61), true change in participant status during the one-month interval could be a plausible source of intra-observer variability in all but one item (global impression of upper limb).

## Discussion

Clinical history taking and assessment are the cornerstones of diagnosis and management [7,10]. Establishing the relevance and reliability of such information is important not only for epidemiological research but also for clinical practice. This study investigated the reliability of two trained observers using a set of standardised questions and assessments derived from a Delphi study and existing literature.

Generally, for clinical interview questions, agreement was high and reliability was good. Reliability for items assessed using measurement instruments and recorded on a numerical scale, for example, grip strength, was generally higher than for items requiring observers to make judgements and interpret participants' responses.

The majority of variables requiring observation and palpation (skin condition, global impression of upper limb, muscle wasting, swelling and pain on resisted movement) showed poor reliability for inter-observer ratings. Reliability was moderate to good for observation and palpation of joint bony change and palpation of joint tenderness, which is similar to findings from previous studies [29,30]. In our study, poor reliability was observed for measurement of thumb opposition (intra-observer), sensory testing and questions relating to altered sensation. Poor reliability may be attributable to several factors.

Real change in symptoms might explain poor reliability, although in this study it is unlikely to explain inter-observer variability. It is more reasonable to expect an effect on intra-observer variability because some change in symptoms over a month (i.e. the period of time between the first and the second assessment) might have occurred. However, the majority of participants reported that their hand symptoms were unaltered, implying a reasonable degree of stability. It should be noted, however, that stability was assessed using a single global question with three response options, and as such conclusions about change in specific symptoms are difficult to draw. Agreement for dimensions likely to change over one month, such as pain, tenderness and swelling, was no poorer for intra- than inter-observer comparisons, suggesting that poor agreement, notably for swelling, was unlikely to be due to change in symptoms.

Order effects are a possible explanation for variability, particularly for inter-observer comparisons of variables that might reasonably improve or deteriorate over the course of the two assessments. The potential for order effects was reduced in the design of the study and no systematic differences were noted when comparing assessors' results for variables likely to change over the course of the assessment.

Poor reliability, particularly for inter-observer ratings, may be explained by systematic differences between the observers. Systematic differences were found between the observers for two of the interview questions relating to altered sensation. Possible explanations for this are that either one of the observers influenced participants in the way in which the question was asked, or the observers interpreted participants" responses differently from each other. Systematic differences were also found for the assessment of muscle wasting, nodes, deformity and swelling with one observer consistently finding more positives than the other. For the assessment of bony enlargement, differences in the number of positive findings were related to the joint group, with one observer finding more enlargements at the proximal interphalangeal (PIP) joints and fewer at the distal interphalangeal (DIP) joints than the other observer. Observers' threshold for making positive judgements may be affected by several factors. Comparative rather than independent judgements may be made within or between participants. Within participants, observers may be influenced in their judgement of the presence of a feature in one joint by what they see in surrounding joints. Similarly, an observers' threshold for judging enlargement or deformity in the joints of one participant may be raised or lowered by judgements made during assessment of previous participants. Despite training the observers using the manual of study protocols, judgements may have been influenced by professional training, post qualification clinical experience, and prior expectation.

In the general population it may be more difficult to differentiate between 'normal' and 'abnormal'. Features in the hand are more likely to be milder and less pronounced than in a secondary care setting, making judgements about their presence more difficult to make, an observation which has been noted previously [30]. For example, in our study, inter and intra-observer reliability for objective testing of sensation using the Semmes-Weinstein™ monofilaments was fair to poor. Our results were similar to those found using healthy volunteers [31,32], but differed from those using nerve injured patients [33,34], where a high degree of reliability was established, suggesting that monofilaments are most reliable for those with definite nerve damage.

High levels of variability, in the face of high observed agreement, may be due to the effects of prevalence, that is, positives occurring either commonly, for example, normal skin condition, or rarely, for example, joint swelling. In these circumstances, a high or low prevalence tends to markedly reduce the magnitude of Kappa, despite high observed agreement. Where prevalence of swelling was not extreme, (notably the index and middle finger metacarpophalangeal joints), reliability was generally better.

Good agreement has previously been observed for the application of the ACR criteria for hand OA [35]. In our study, the observers demonstrated moderate reliability when applying the ACR criteria for hand OA. This slight difference may be due to variations in the two study populations.

Poor reliability is likely to be due to a combination of differences between the observers, features in the hand being indistinct in nature, and a high or low prevalence of features. The reliability of assessing items such as altered sensation may benefit from greater standardisation or alternative forms of data collection, for example, self-report questionnaire. The reliability of assessment of individual features at single joints, for example, nodes, may benefit from being viewed in combination for composite variables, cut-offs, or classifications. These results suggest that the ACR criteria for hand OA is more reliable than the individual components.

In the absence of accepted gold standards for assessing specific patient populations [36], it is difficult to comment on the accuracy of the observers' judgements. Where there was agreement between observers it does not necessarily mean that the answer is correct [37]. Similarly, where there was systematic disagreement, it is difficult to say which of the observers was correct.

This reliability study has several strengths. The questions and assessments were derived from Health Care Professional consensus [11], supplemented by measures from the literature. Participants were sampled purposively from a primary care setting to ensure a broad spectrum of hand problem severity. Potential sources of variability were minimised through observer training and the use of standardised protocols and aid memoirs. The potential for order effects was reduced in the design of the study. The time interval between repeat assessments was chosen to ensure a balance between participants remembering details of the assessment and true change occurring.

It has been acknowledged that there is no single design that would adequately address issues of external validity for method, measuring instruments, observers and participants [38]. Whereas this study focused on ensuring external validity in relation to participants, the results based on two observers will limit the extent to which generalisations about the reliability related to the wider population of clinicians can be made [39].

Although this study was designed to limit potential sources of variability, it is inevitable that some bias occurred. Systematic differences between observers may be responsible in part for some of the poor reliability achieved, and could be addressed to an extent by further training, strengthening of study protocols, and routine quality control checks to ensure adherence to protocols. However, it is inevitable that when making judgements, particularly about the presence of mild features, some variation will occur [39].

## Conclusions

This study has established the reliability of two trained observers from different professional backgrounds administering clinical interview questions and assessing the hands of 55 community-dwelling older adults with self-reported hand problems. The findings from this study suggest that whilst the majority of clinical interview questions and some of the hand assessment variables were reliable, others were not. Further training and strengthening of protocols may help to reduce systematic differences between observers and improve agreement.

In light of poor reliability for some items occurring mainly due to a combination of low prevalence of features and systematic differences between the observers, the decision to use clinical interview and hand assessment variables in clinical practice or further research in primary care should include consideration of clinical applicability and training alongside reliability.

Further investigation is required to determine the relationship between these clinical questions and assessments and the clinical course of hand pain and hand problems in community-dwelling older adults.

## Competing interests

None of the authors has any financial or other relationships that might lead to a conflict of interest.

## Authors' contributions

All authors contributed to the conception and design, execution, analysis and interpretation of data, were involved in drafting and critically revising the article, and read and approved the final version.

## Pre-publication history

The pre-publication history for this paper can be accessed here:

http://www.biomedcentral.com/1471-2474/12/3/prepub
